# The roles of ING5 expression in ovarian carcinogenesis and subsequent progression: a target of gene therapy

**DOI:** 10.18632/oncotarget.21968

**Published:** 2017-10-19

**Authors:** Hua-Chuan Zheng, Shuang Zhao, Yang Song, Xiao-Qing Ding

**Affiliations:** ^1^ Department of Experimental Oncology and Animal Center, Shengjing Hospital of China Medical University, Shenyang 110004, China; ^2^ Department of Pathology, The First Affiliated Hospital of Jinzhou Medical University, Jinzhou 121001, China

**Keywords:** ovarian cancer, ING5, pathogenesis, progression, prognosis

## Abstract

Here, we found that ING5 overexpression suppressed cell viability, glucose metabolism, migration, invasion and epithelial-mesenchymal transition, and induced cell arrest, apoptosis, senescence, autophagy and fat accumulation in ovarian cancer cells. ING5-mediated chemoresistance was positively linked to apoptotic resistance and chemoresistance-related gene expression. ING5 overexpression suppressed tumor growth of ovarian cancer by decreasing proliferation, and inducing apoptosis and autophagy. ING5 mRNA level was lower in ovarian cancer than normal ovary, and borderline than benign tumors (*p* < 0.05), and negatively correlated with vascular invasion, lymphatic invasion and FIGO staging of ovarian cancer (*p* < 0.05). ING5 protein was less expressed in primary cancer than normal ovary (*p* < 0.05). There was a negative correlation between *ING5* mRNA expression and the overall or progression-free survival time of the cancer patients with Grade 2, Grade 3, and stage I cancer (*p* < 0.05). Immunohistochemically, ING5 was less expressed in serous and mucinous adenocarcinoma than miscellaneous subtypes, and positively correlated with dedifferentiation and ki-67 expression of ovarian cancer (*p* < 0.05). These data suggested that down-regulated ING5 expression might be involved in ovarian carcinogenesis possibly by suppressing aggressive phenotypes, including proliferation, tumor growth, migration, invasion, and anti-apoptosis.

## INTRODUCTION

Inhibitor of growth 5 (ING5) belongs to the encoding protein of Class II tumor suppressor gene because its inactivation results from frequent genetic and epigenetic alterations. Structurally, it includes LZL (leucine zipper like), NCR (novel conserved region), NLS (nuclear localization signal), and PHD (plant homeo domain) from N-terminal to C-terminal. ING5 might promote DNA repair, and induce apoptosis and chromatin remodeling by forming histone acetyl transferase (HAT) complexes [1–5]. ING5 was reported to activate the cyclin-dependent kinase inhibitor p21/waf1 promoter to induce p21/waf1 expression, enhance p53 acetylation at Lys-382 and Lys-120 residues, and physically interact with p300, a member of HAT complexes, and p53 *in vivo* [6].

ING5 overexpression can decrease colony-forming efficiency and cell population in S phase, and induce apoptosis in a p53-dependent manner [6]. Recently, down-regulated expression of *ING5* mRNA was detectable in head and neck squamous cell carcinoma (HNSCC) with missense mutations located within LZL finger and NCR domains of ING5 protein [7]. The hypoexpression and nucleocytoplasmic translocation of ING5 protein were observed in HNSCC [8], gastric [9], colorectal [10] and lung [11] cancers, and positively associated with their aggressiveness. ING5 overexpression might reverse the aggressive phenotypes of gastric, breast and lung cancer cells, such as proliferation, migration, invasion, epithelial-mesenchymal transition (EMT), growth or metastasis [11–13]. However, our findings indicated that ING5 overexpression might activate β-catenin, Akt and NF-κB pathways in SGC-7901 cells, and increase the apoptotic and chemotherapeutic resistances [12]. ING5 overexpression increased glycolysis and subsequent aerobic oxidation of lung cancer cells, which was closely linked to PFK-1 and PDPc overexpression. Additionally, aberrant fat accumulation in ING5 transfectants might be attributable to the up-regulatory ADFP expression [13].

Ovarian cancer is the second leading cancer and the 5th leading cause of cancer-related deaths in women. The five-year survival rate of ovarian cancer is only 47% because no sophisticated approach for the early diagnosis makes most ovarian cancers diagnosed at advanced stages [14, 15]. To identify novel biomarkers for cancer diagnosis and novel targets for treatment, we observed the effects of ectopic ING5 overexpression on the aggressive phenotypes of ovarian cancer cells, and analyzed the relevant mechanisms. *ING5* expression was examined in ovarian cancer, and compared with the clinicopathological parameters to explore the roles of ING5 expression.

## RESULTS

### The effects of ING5 on proliferation and apoptosis of ovarian cancer cells

**Figure 1:**
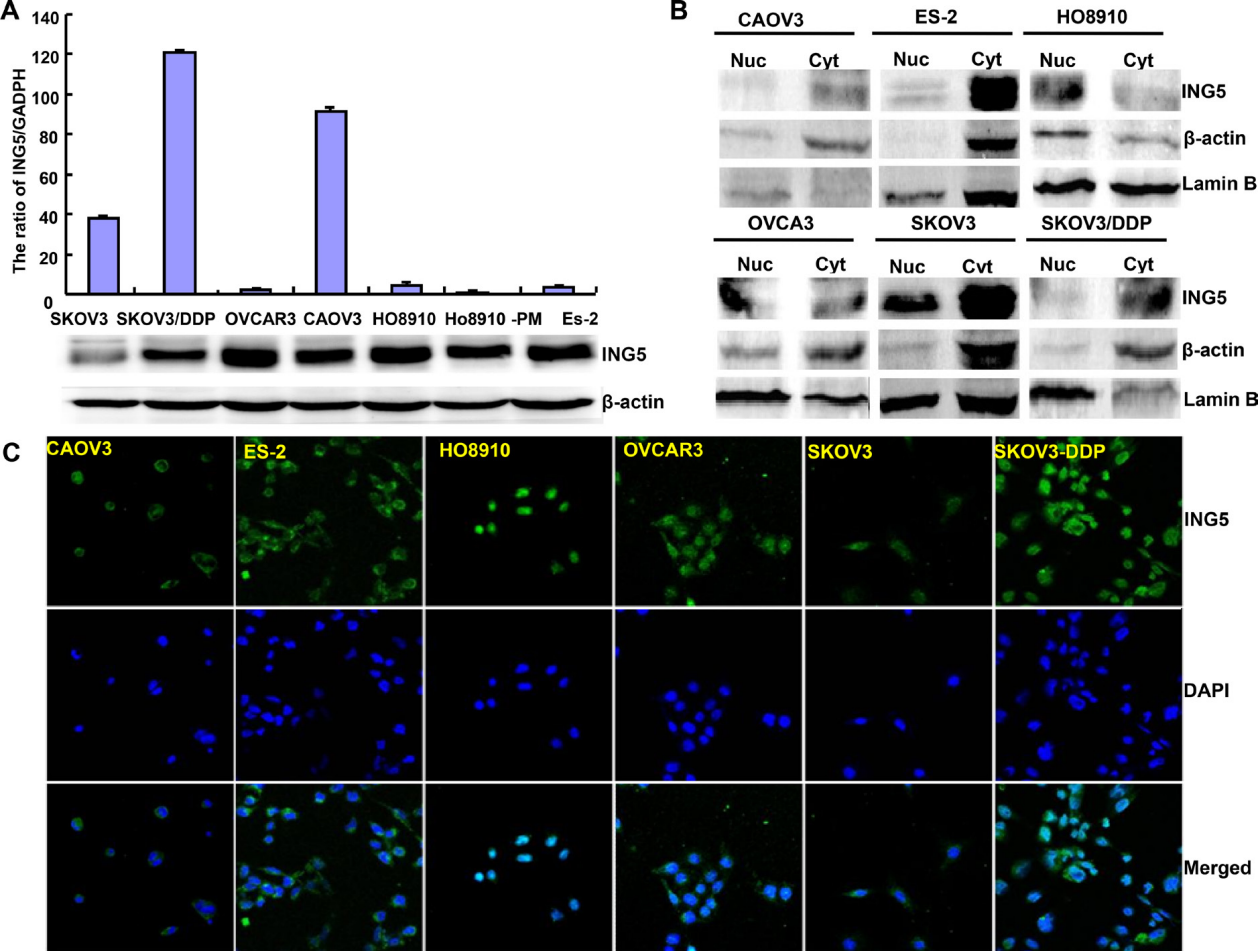

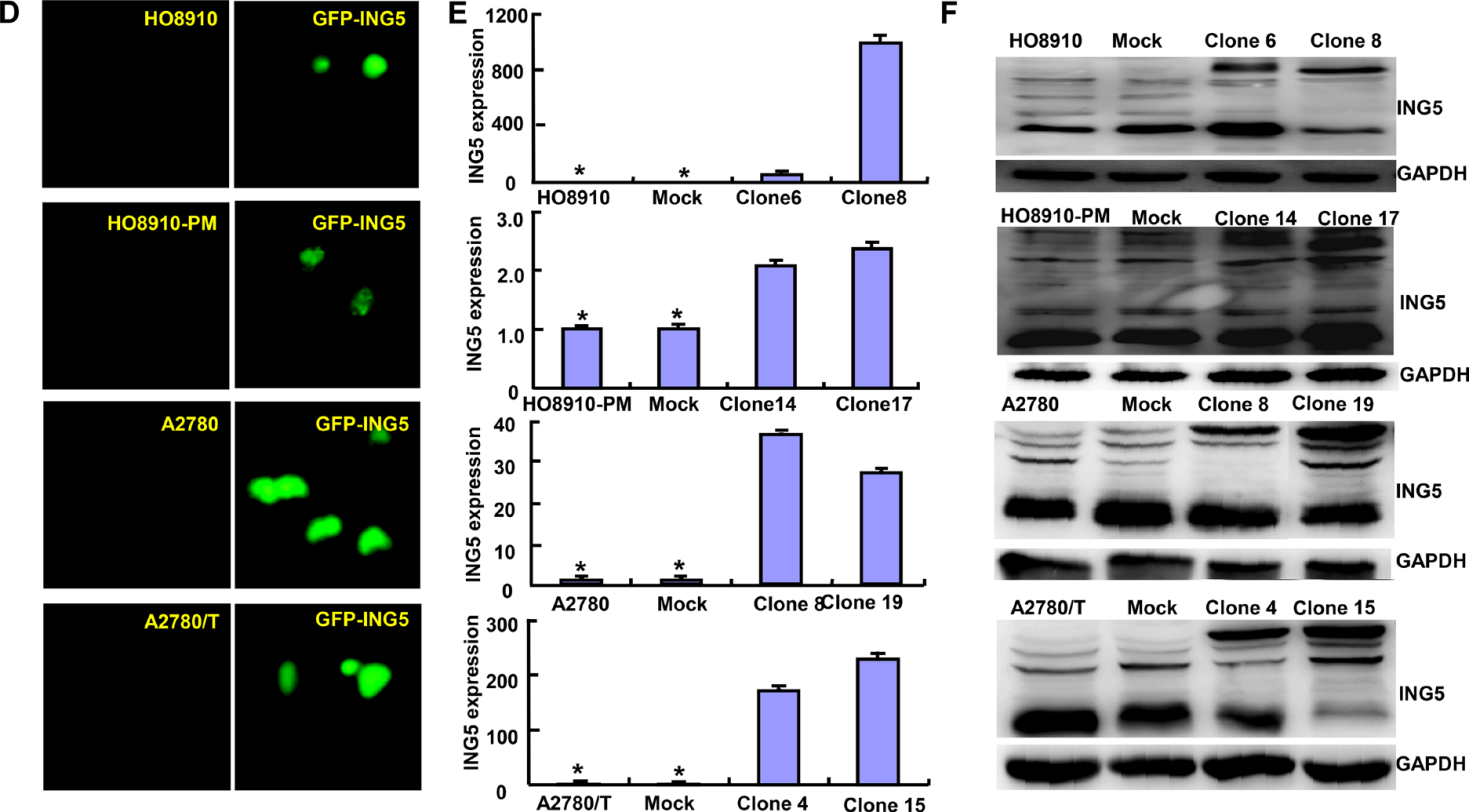
The expression and subcellular localization of ING5 in ovarian cancer cells Endogenous ING5 expression was screened in several kinds of ovarian cancer cells, including SKOV3, SKOV3/DDP, OVCAR3, OVCA3, HO8910, HO8910-pm, and ES-2, evidenced by RT-PCR (**A**), Western blot (A, **B**), immunofluorescence and nucleocytosolic fraction (**C**). After transfection of pEGFP-N1-ING5, ING5 expression became strong in HO8910, HO8910-PM, A2780 and A2780/T cells by immunofluorescence (**D**), RT-PCR (**E**), and Western blot (**F**). ^*^p < 0.05, compared with transfectants.

At the protein level, ING5 was lowly expressed in SKOV3 in comparison to the other cells (Figure 1A). Immunofluorescence and nucleocytosolic fraction showed that ING5 expression was observed in nucleus of HO8910, in the nucleus and cytoplasm of OVCAR3, while in the cytoplasm of ES-2, SKOV3 and SKOV3/DDP (Figure 1B and 1C). We successfully transfected its expressing plasmid into HO8910, HO8910-PM, A2780 and A2780/T cells, evidenced by GFP tag, real-time RT-PCR and Western blot (Figures 1D–1F).

**Figure 2:**
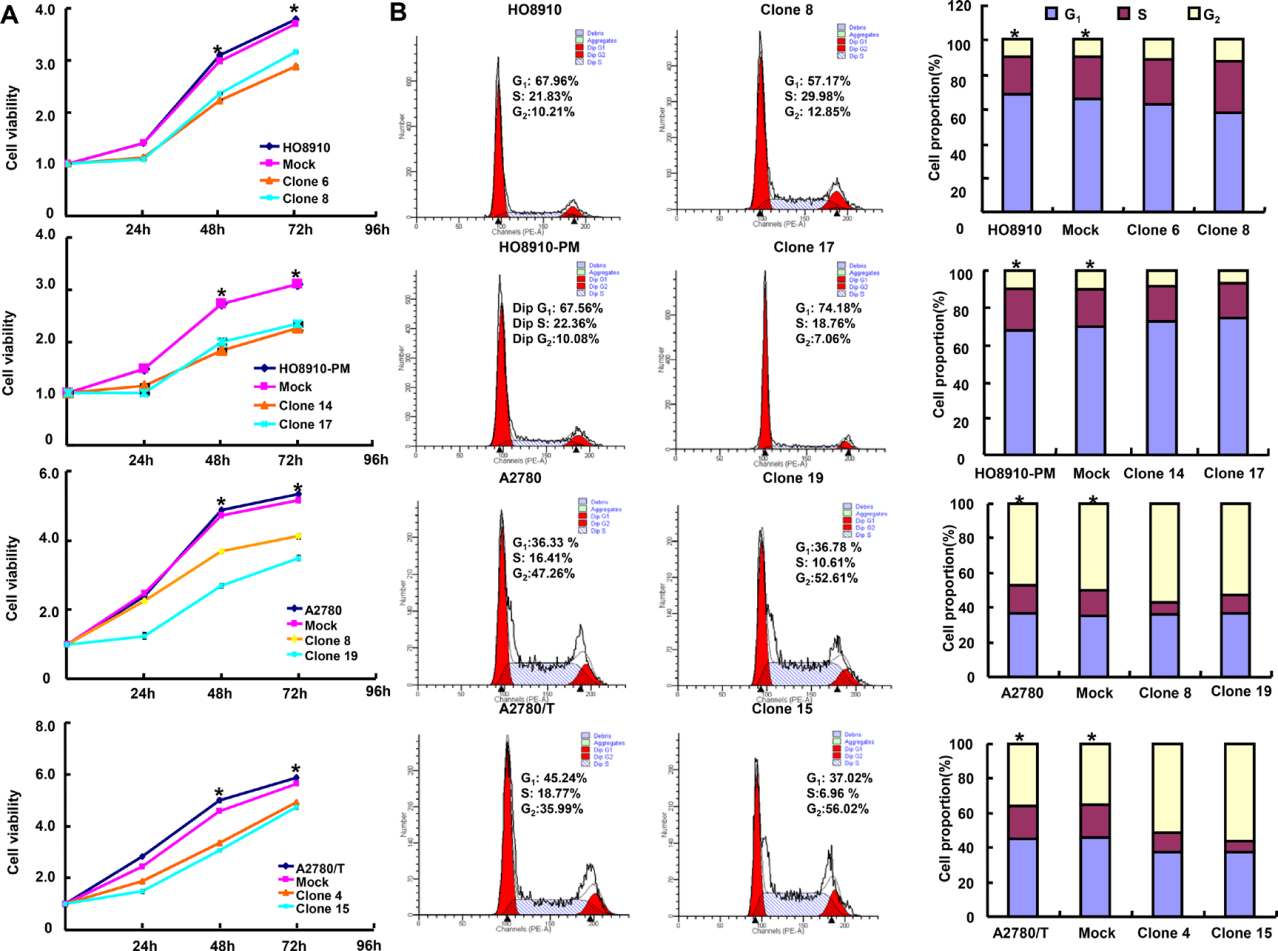

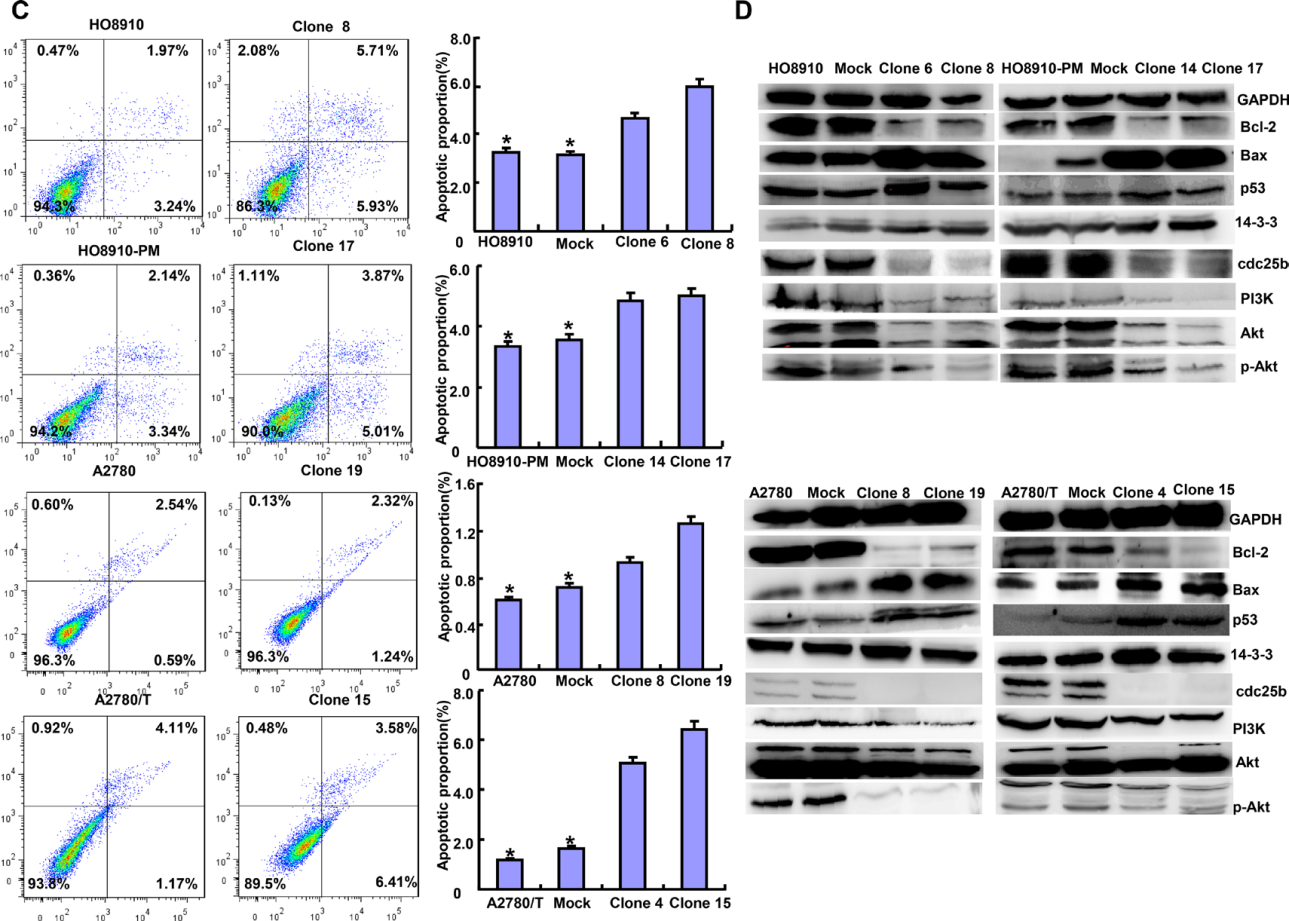
The effects of ING5 on apoptosis and proliferation of ovarian cancer cells The transfectants showed a lower growth in comparison with the control and mock (**A**). Ectopic ING5 expression could induce G_2_ arrest of HO8910, A2780 and A2780/T transfectants by PI staining, while G_1_ arrest of HO8910-PM transfectants (**B**). The transfectants showed a high apoptosis, evidenced by Annexin V assay (**C**). The apoptosis-related and cell-cycle-related proteins were screened by Western blot (**D**). ^*^p < 0.05, compared with transfectants.

ING5 overexpression reduced cell viability and induced apoptosis of ovarian cancer cells than the control and mock (Figures 2A and 2C, *p* < 0.05). It caused G_2_ arrest in HO8910, A2780 and A2780/T cells, and G_1_ arrest in HO8910-PM (Figure 2B*, p* < 0.05). In ING5 transfecants, there appeared the overexpression of p53, 14-3-3 and Bax, whereas the hypoexpression of cdc25b, Bcl-2, PI3K, Akt and p-Akt in ovarian cancer cells by western blot (Figure 2D).

### The effects of ING5 on invasion and migration of ovarian cancer cells

**Figure 3:**
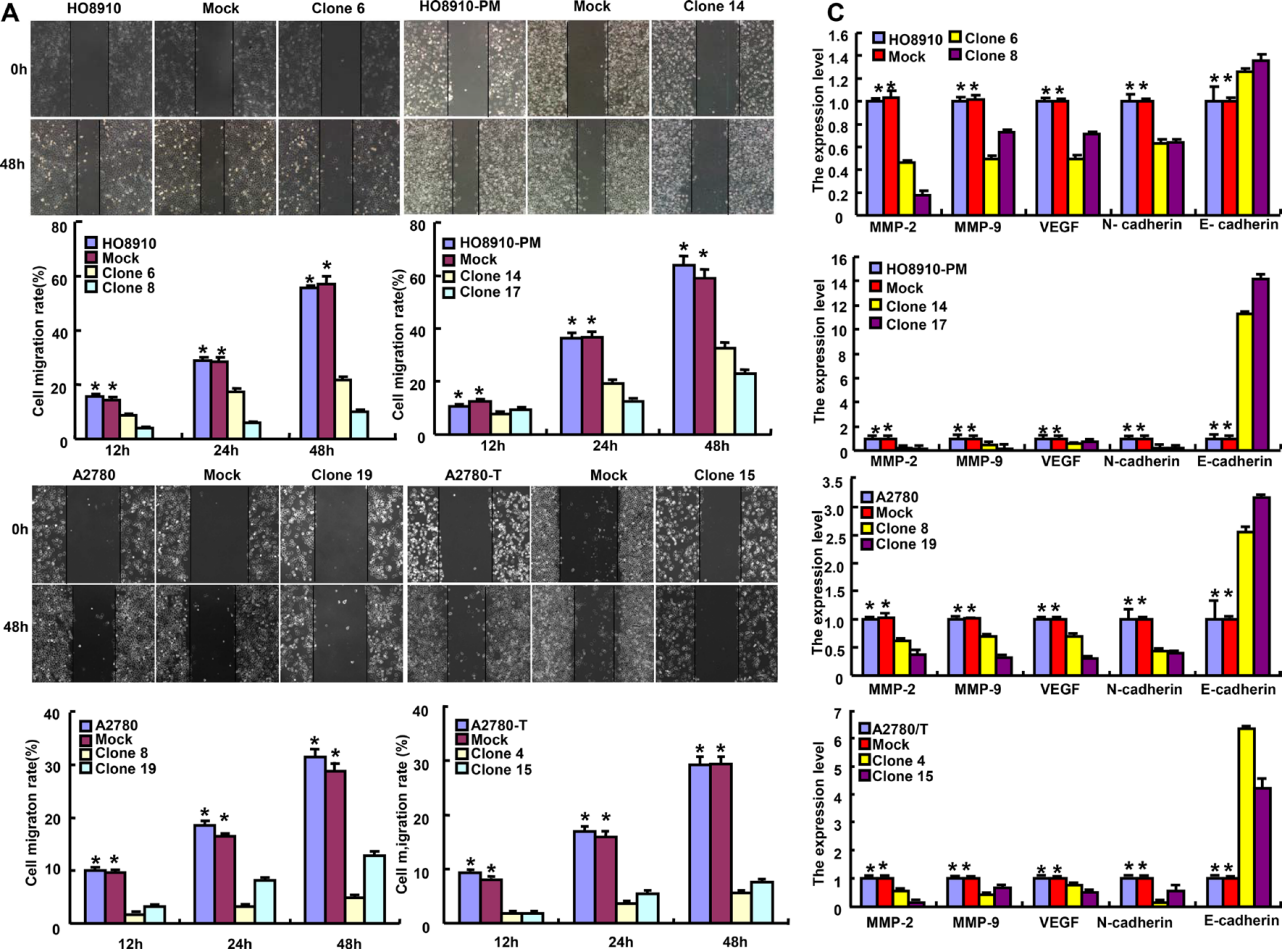

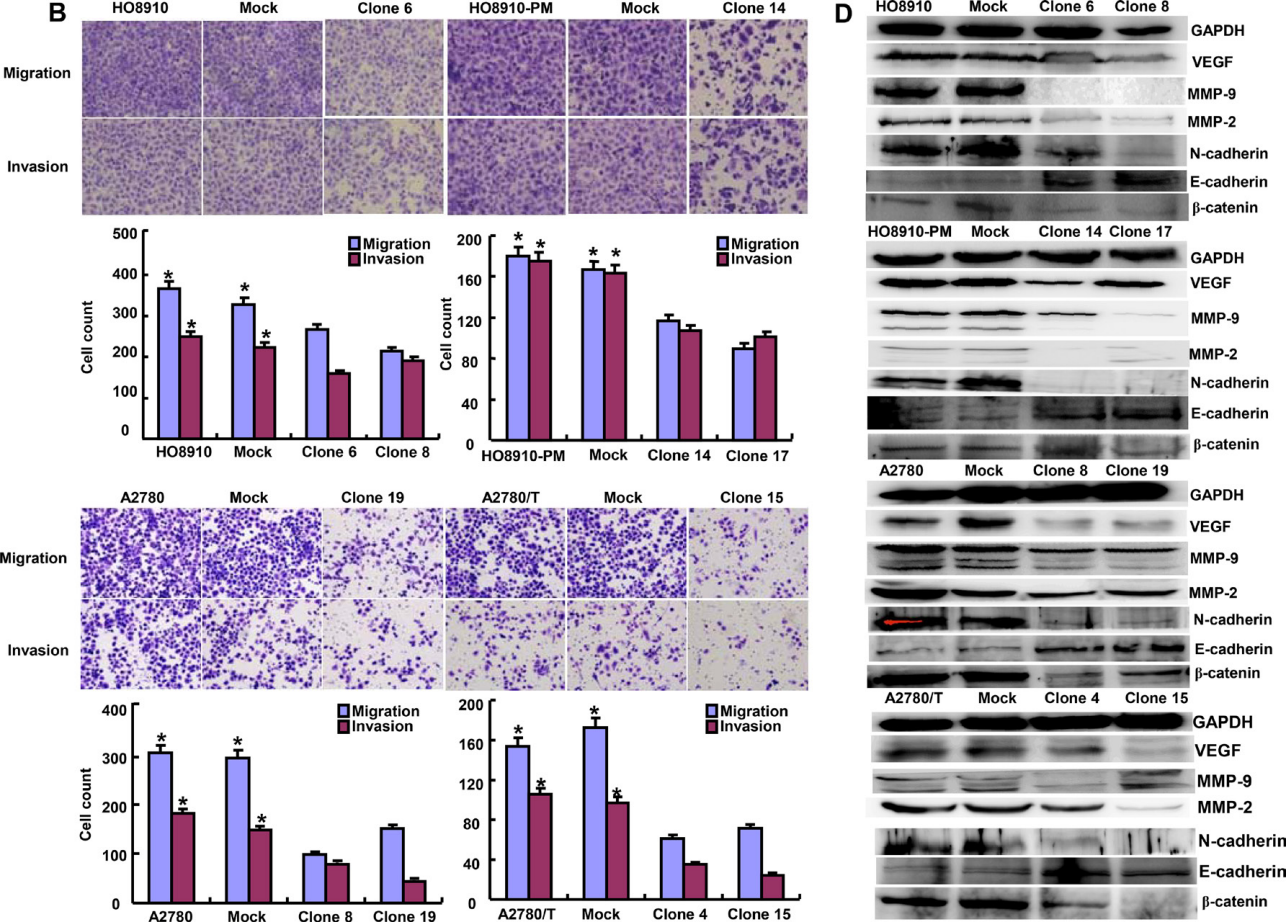
The effects of ING5 on invasion and migration of ovarian cancer cells ING5*-*overexpressing cells had a weaker ability in invasion and migration than the control and mock according wound healing **(A)** and transwell chamber assay **(B)**. The expression of phenotype-related molecules was screened by real-time RT-PCR **(C)** and Western blot (**D**). ^*^p < 0.05, compared with transfectants.

Based on wound healing and transwell chamber assay, cell migration and invasion was weakened in ING5 transfectants (Figures 3A and 3B, *p* < 0.05). At the levels of protein and mRNA, ING5 overexpression decreased the expression of VEGF, MMP-2 and MMP-9 in transfectants (Figures 3C and 3D, *p* < 0.05). Interestingly, N-cadherin expression was decreased in transfectants in comparison to the control and mock, while versa for E-cadherin (Figures 3C and 3D). The lower expression of β-catenin was seen in ING5 transfectants than control and mock (Figure 3D).

### The effects of ING5 on senescence, autophagy and metabolism of ovarian cancer cells

**Figure 4:**
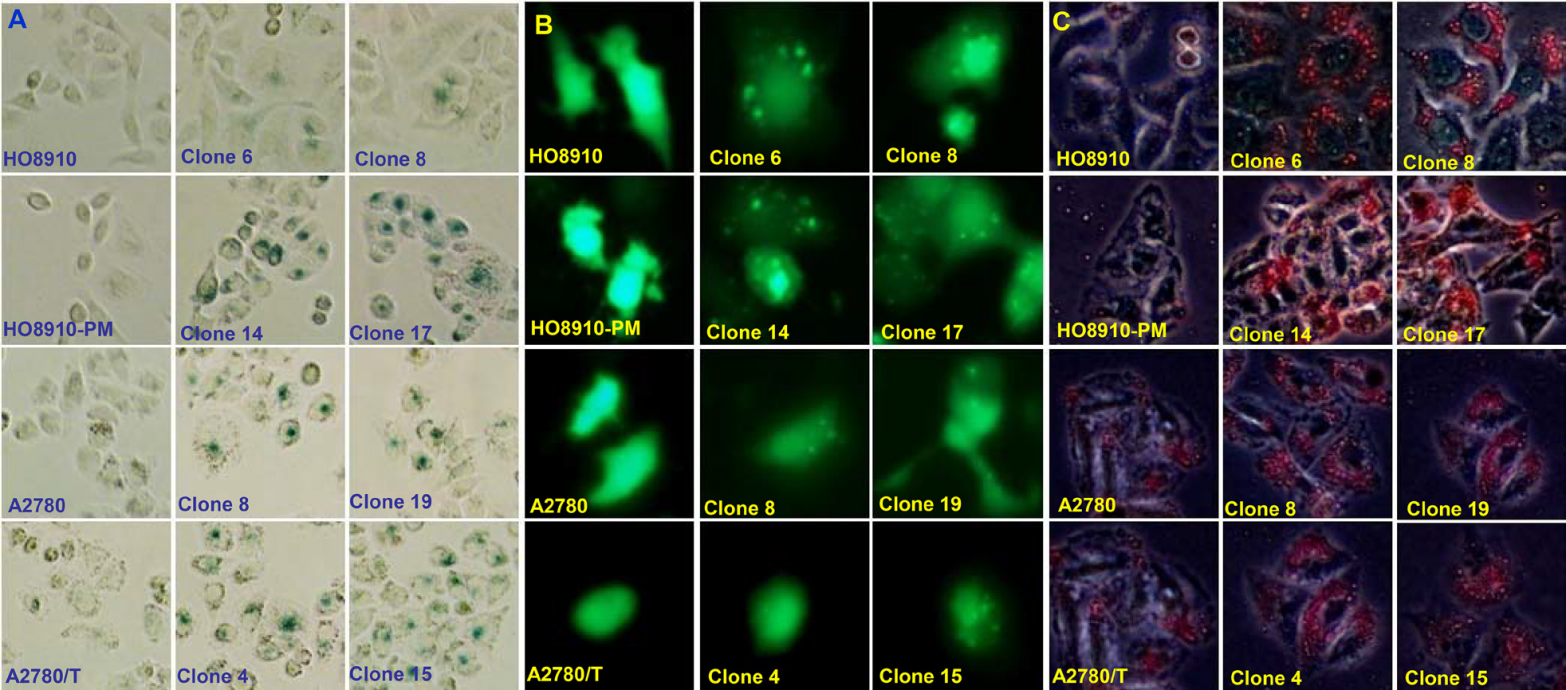

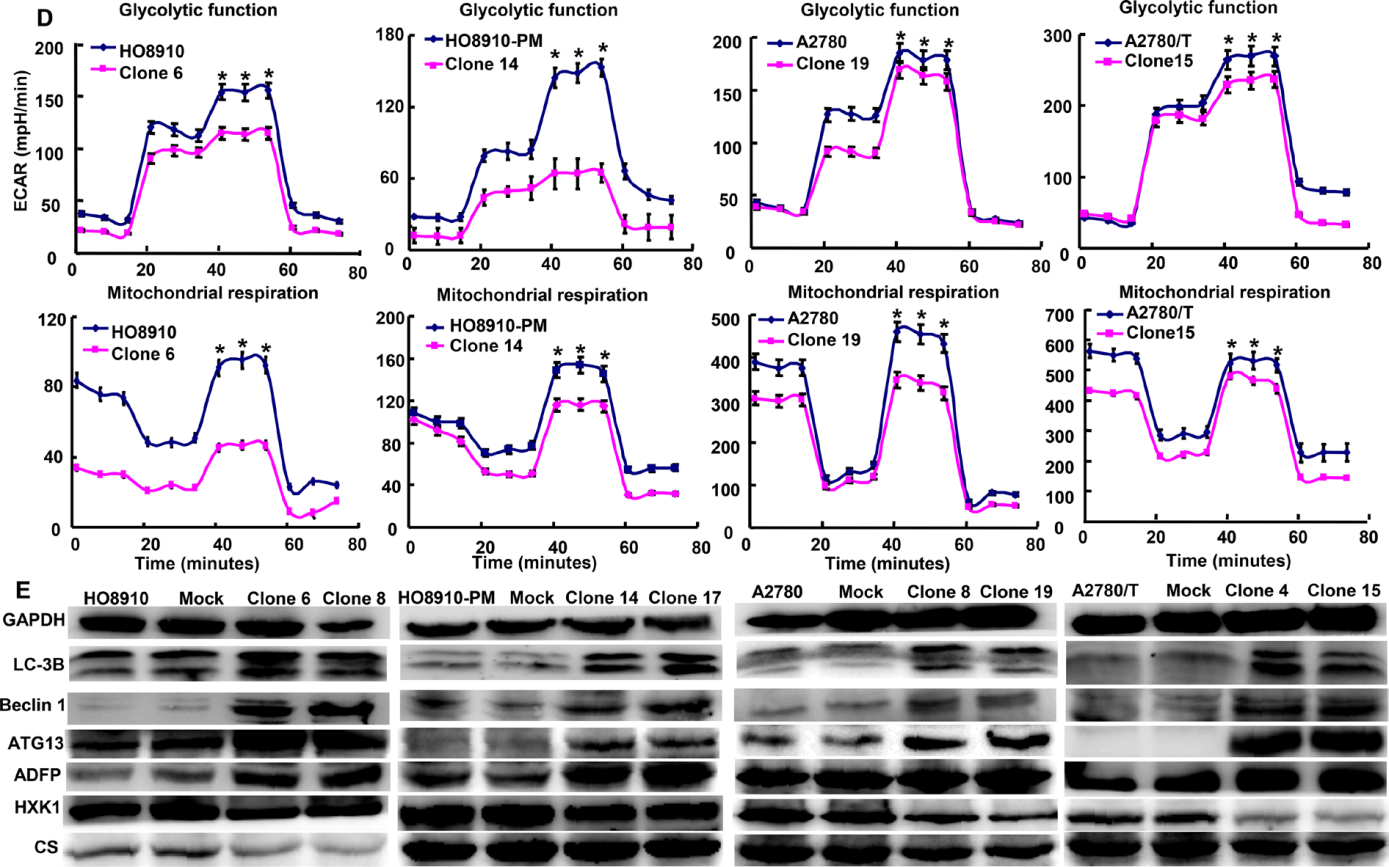
The effects of ING5 on senescence, autophagy and metabolism of ovarian cancer cells ING5 transfectants showed a higher senescence evidenced by galactosidase staining (**A**), and a higher autophagy by the transient transfection of GFP-tagged LC-3B plasmid (**B**) and aberrant fat accumulation by oil red O staining (**C**). All ING5 transfectants showed a lower glycolytic metabolism, and mitochondrial respiration, evidenced by measurement of extracellular acidification rate and oxygen consumption rate (**D**). The phenotype’ proteins were examined by Western blot (**E**). ^*^p < 0.05, compared with transfectants.

A higher number of β-galactosidase-positive cells were observed in ING5 transfectants than the control (Figure 4A). ING5 overexpression induced autophagy according to the morphological appearance of ovarian cancer cells after the transient transfection of GFP-tagged LC-3B plasmid (Figure 4B). According to oil red O staining, ING5 transfectants showed a high fat accumulation (Figure 4C). ING5 transfectants showed lower glycolytic metabolism mitochondrial respiration than their parental cells (Figure 4D, *p* < 0.05). ING5 increased the expression of LC-3B, Beclin 1, ATG13 and ADFP, but decreased the expression of HXK1 and CS (Figure 4E).

### The correlation between ING5 and the chemosensitivity of ovarian cancer cells

**Figure 5:**
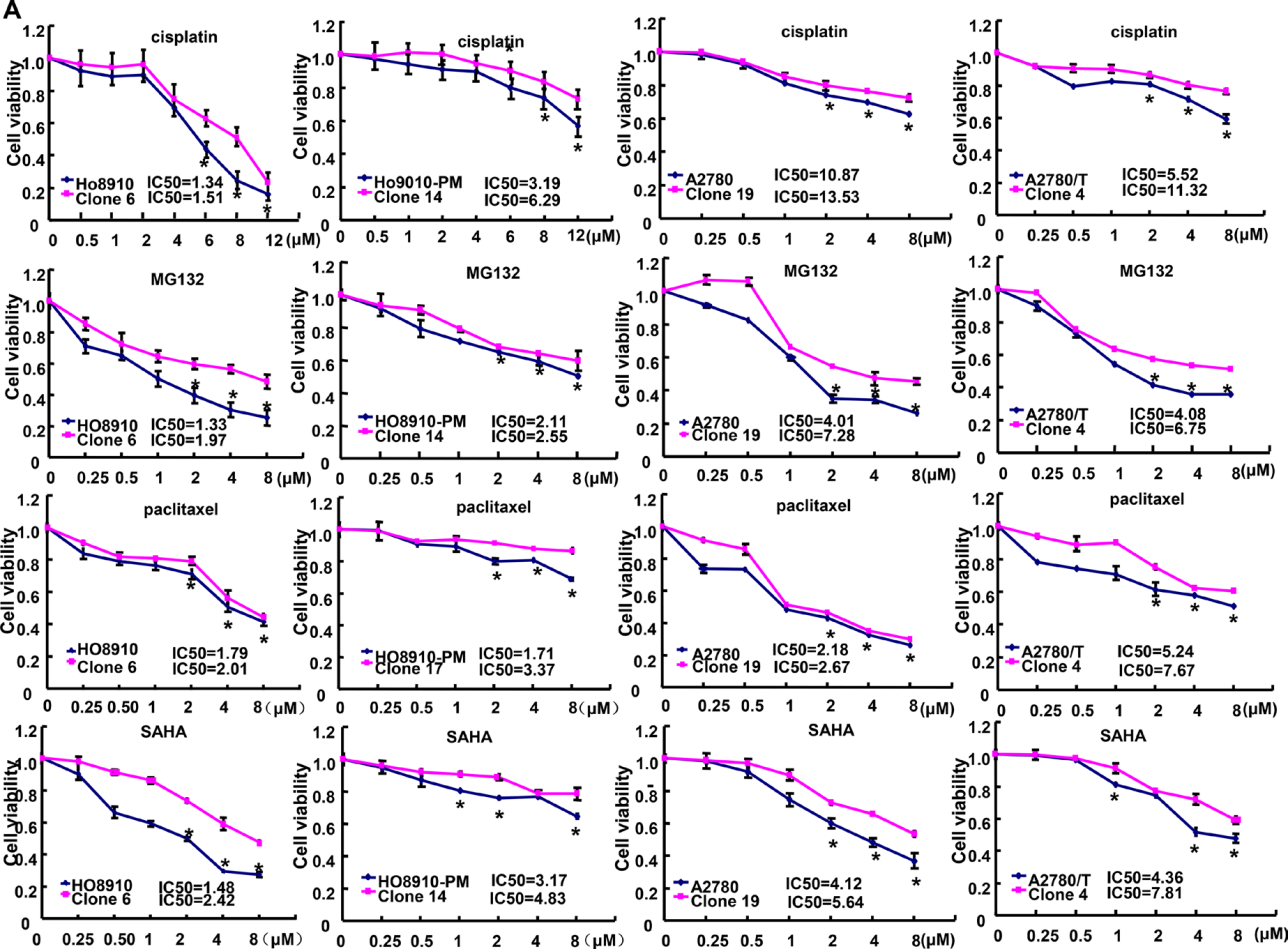

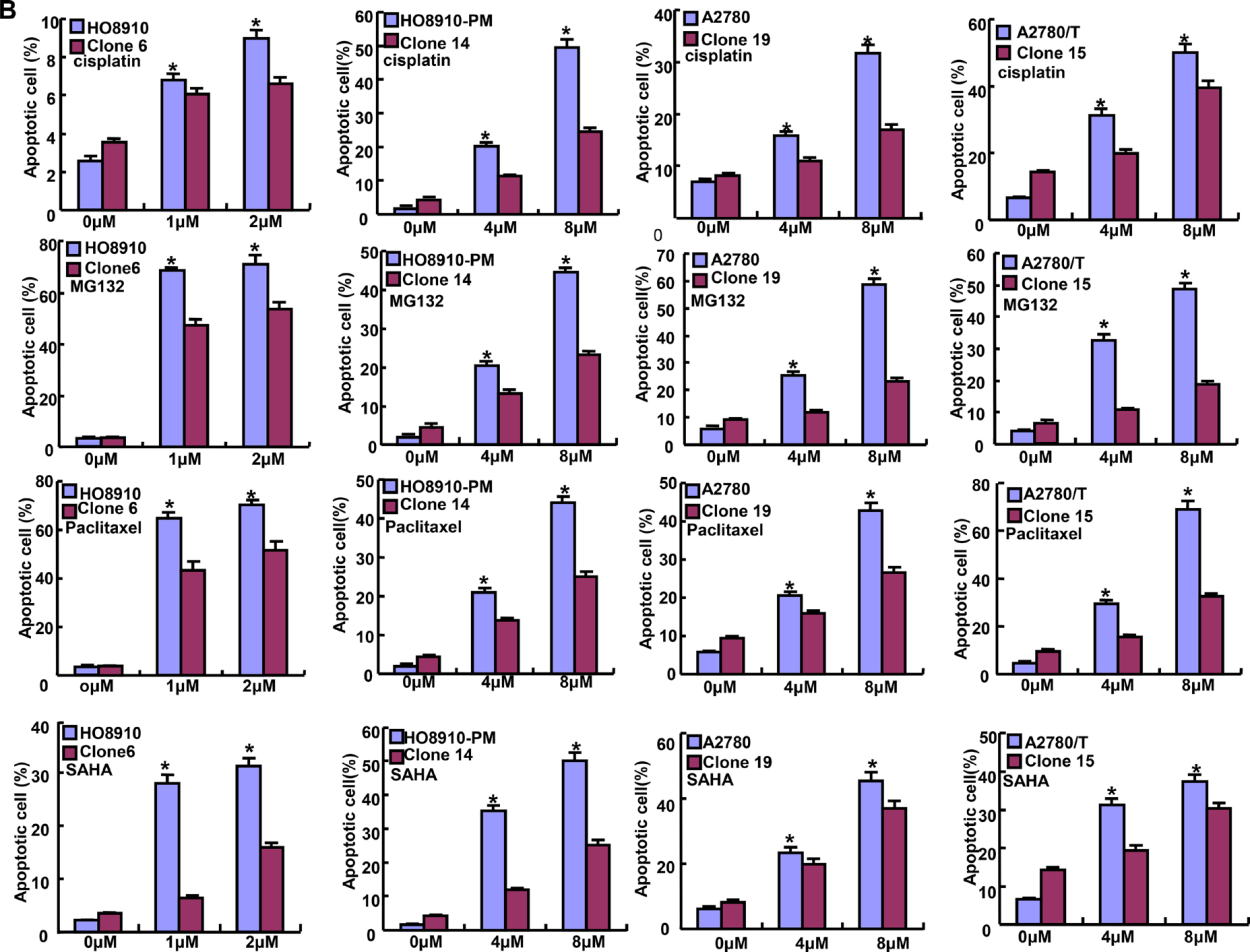

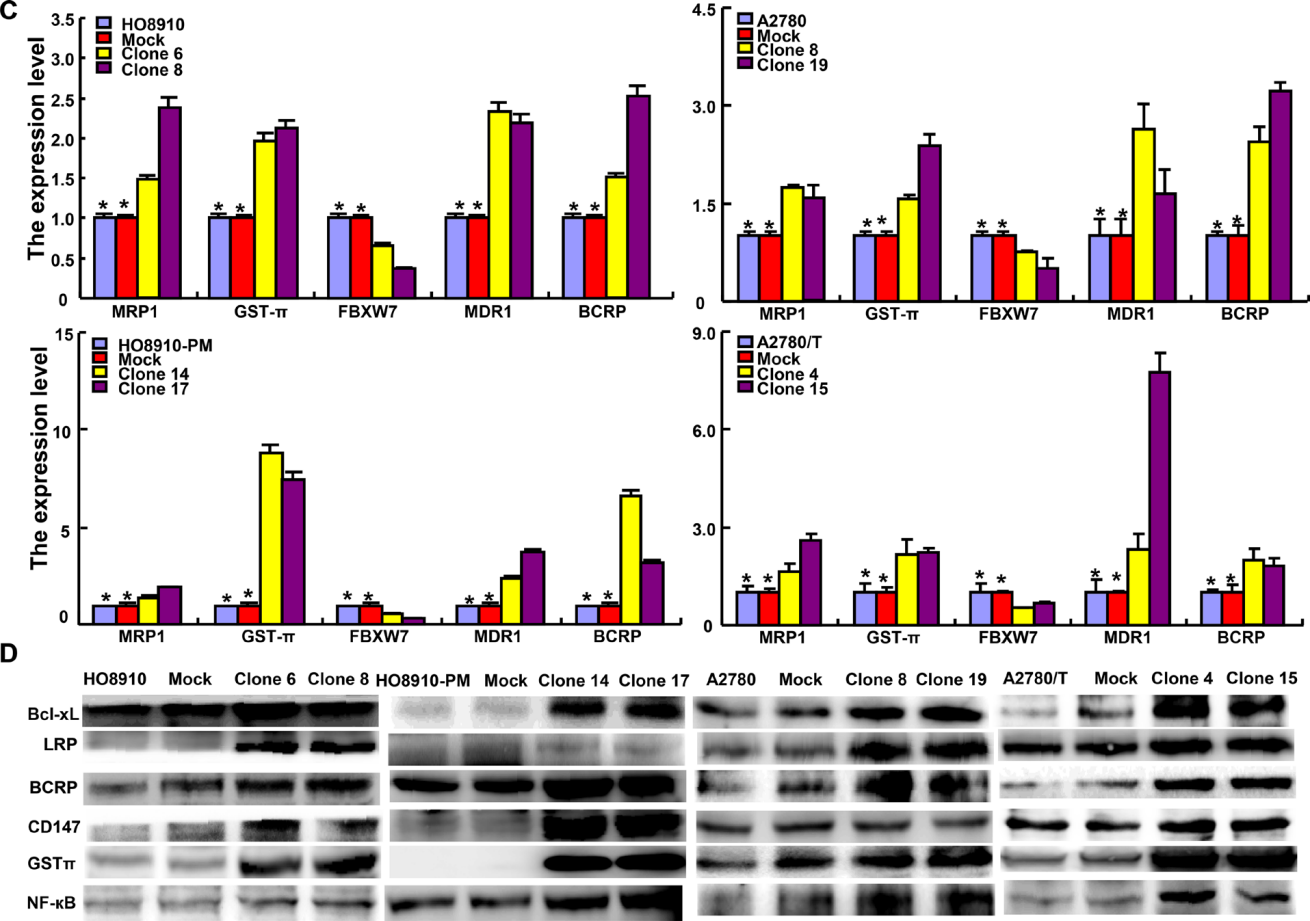
The correlation between ING5 and the chemosensitivity of ovarian cancer ING5 transfectant showed a higher sensitivity to cisplatin, MG132, paclitaxel and SAHA than the control by MTT assay (**A**) and lower level of apoptotic induction by Annexin V (**B**). The chemoresistance-related molecules were screened by real-time RT-PCR (**C**) and Western blot (**D**). ^*^p < 0.05, compared with transfectants.

After exposed to cisplatin, MG132, paclitaxel and SAHA, *ING5* transfectants showed higher viability and lower apoptosis than the control in both time- and dose-dependent manners, evidenced by CCK-8 assay and FASC assay respectively (Figures 5A and 5B, *p* < 0.05). In addition, we found that mRNA expression of FBXW7 was down-regulated, whereas GST-π, MRP1, MDR1 and BCRP were up-regulated in both transfectants, compared with the control and mock (Figure 5C, *p* < 0.05). There appeared a higher expression of Bcl-xL, LRP, BCRP, CD147, GST-π and NF-кB in ING5 transfectans than the control and mock (Figure 5D).

### The inhibitory effects of ING5 on the tumor growth of ovarian cancer cells

**Figure 6:**
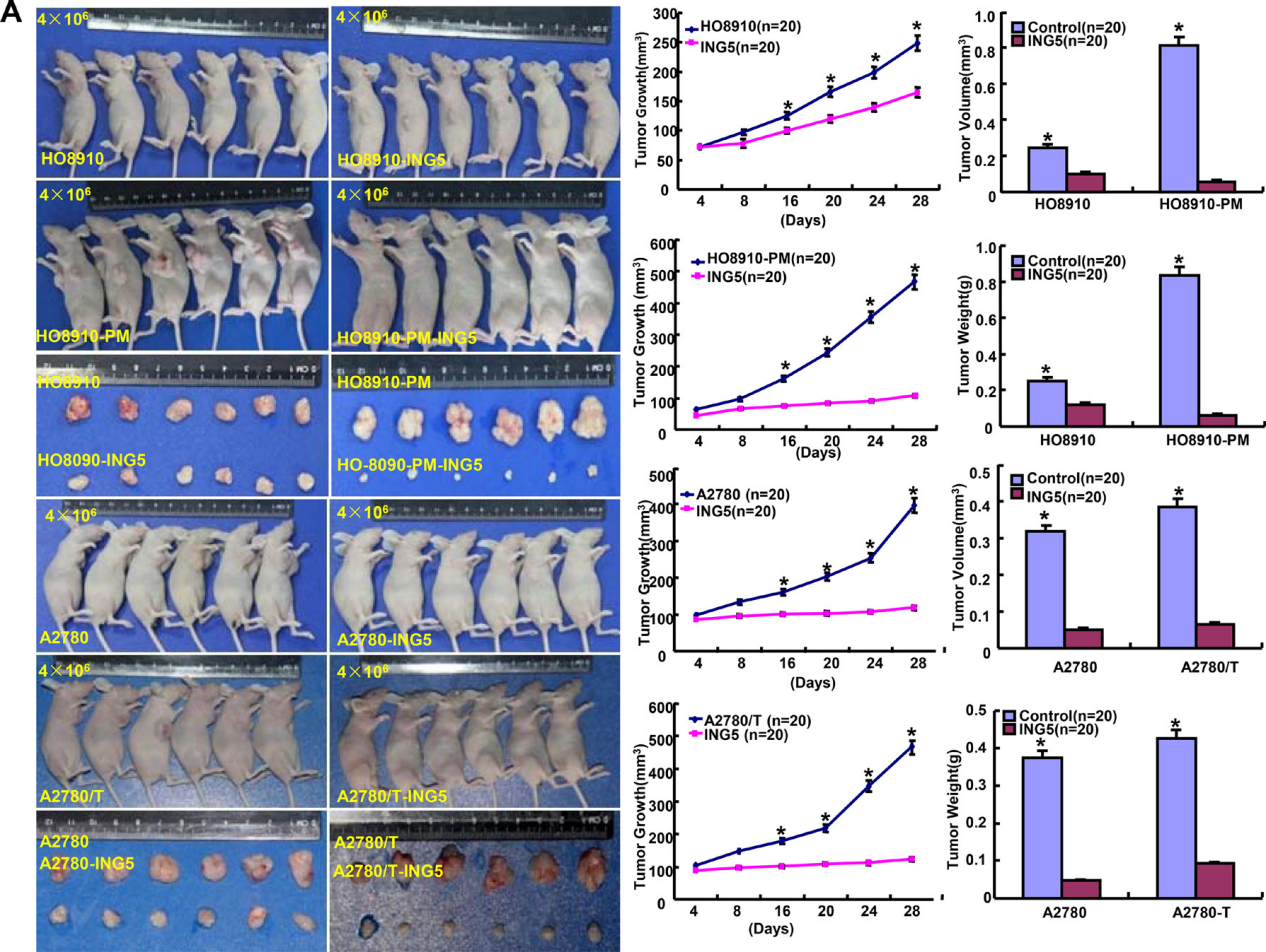

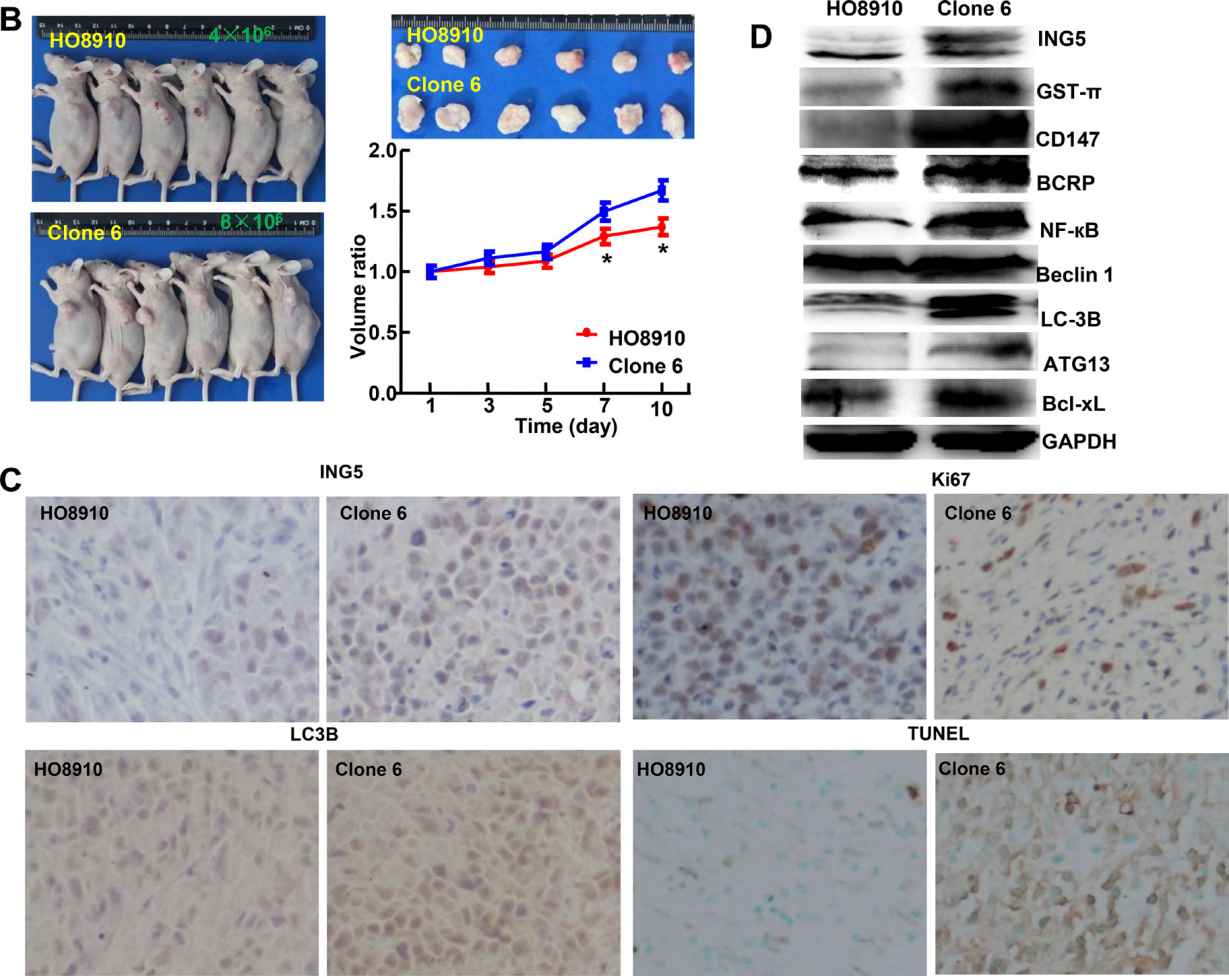
The effects of ING5 on the tumor growth of ovarian cancer cells The growth of four ovarian cancer cells (HO-8910, HO-8910/pm, A2780 and A2780/T) was faster than their ING5 tranfectants by measuring tumor volume and weight (**A**). The growth rate of HO8910 cells was lower than their ING5 transfectants after treatment with SAHA (**B**). The transfectant cells showed stronger ING5 expression, weaker ki-67 expression, higher LC3B immunoreactivity and higher signals of TUNEL than the control (**C**). The chemoresistance-related proteins were detected by Western blot (**D**). ^*^p < 0.05, compared with transfectants.

We subcutaneously transplanted ovarian cancer cells and their ING5 transfectants into nude mice, and found that the tumor volume and weight of ING5 transfectants were larger than the control by rule measurement, calculation and weighting (Figure 6A, *p* < 0.05). After the exposure to SAHA, the growth rate was lower in HO8910 than its ING5 transfectants (Figure 6B). ING5 transfectants showed lower proliferation, higher apoptosis and autophagy than the control, evidenced by Ki-67 and LC3B immunostaining, and TUNEL respectively (Figure 6C). According to Western blot, the expression of ING5, Bcl-xL, BCRP, CD147, Beclin 1, ATG13, LC-3B, GST-π and NF-кB was up-regulated in ING5 transfectans in comparison to the control (Figure 6D).

### The correlation of ING5 expression with the aggressiveness of ovarian cancer

**Figure 7:**
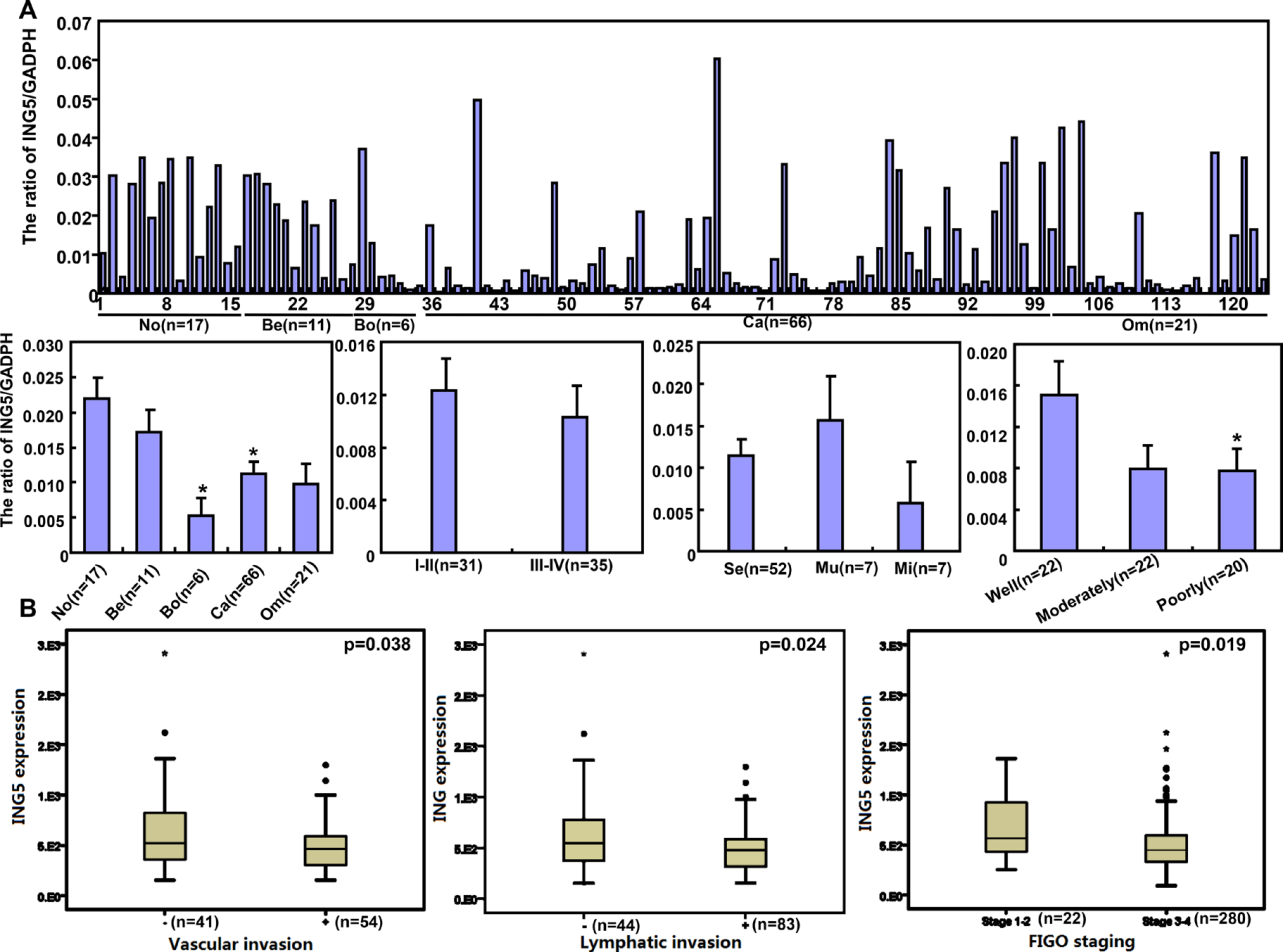

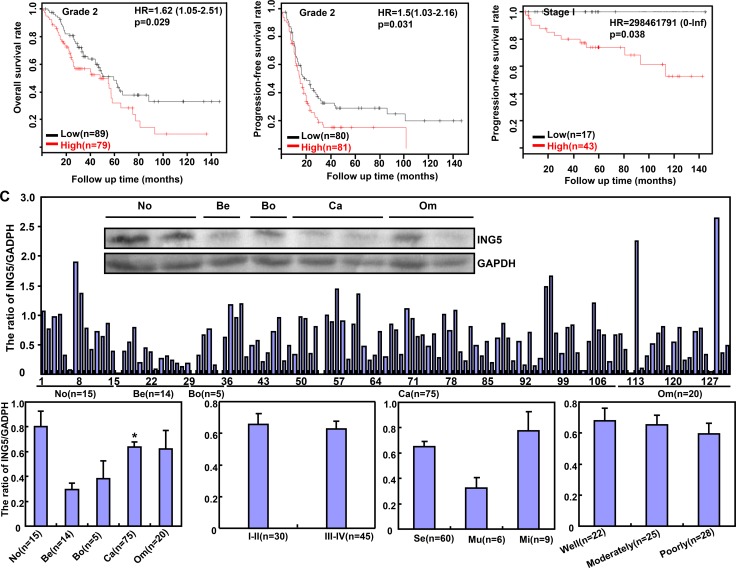

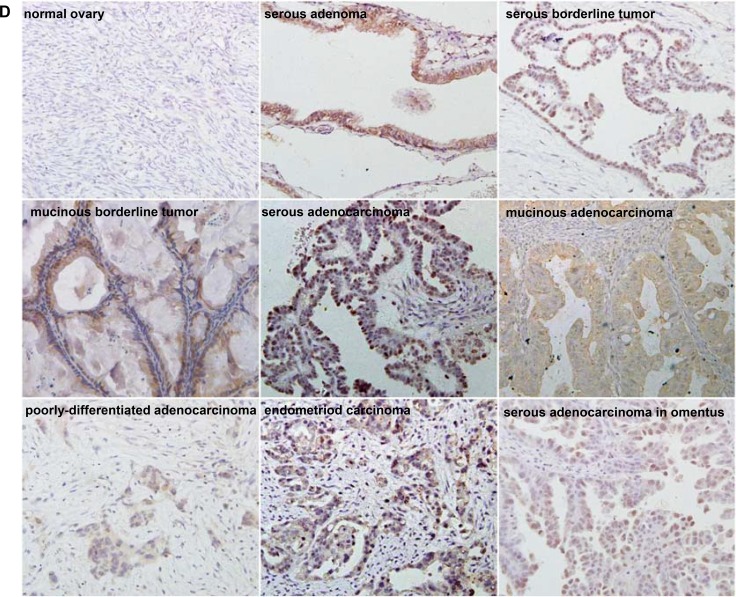
The clinicopathological significances of ING5 expression in ovarian cancers *ING5* mRNA was quantified in ovarian normal tissue (No), benign (Be) and borderline (Bo) tumor, primary cancer (Ca), and metastatic cancer in omentum (Om) by real-time PCR with its correlation between its expression and clinicopathological features analyzed (**A**). The clinicopathological and prognostic significances of *ING5* mRNA expression were analyzed using TCGA dataset and KM plotter respectively (**B**). Tissue lysate was loaded and probed with anti-ING5 antibody with GAPDH as an internal control by Western blot. ING5 protein was examined in No, Be, Bo, Ca and Om, and compared with aggressive parameters (**C**). Additionally, ING5 was not expressed in normal fiber cells, but strongly in the nucleus and cytoplasm of serous adenoma, serous and mucinous borderline tumor, serous, mucinous, and poorly-differentiated adenocarcinoma, endometriod carcinoma, serous adenocarcinoma in omentus by immunohistochemistry (**D**). The data is expressed as mean ± standard error. ^*^p < 0.05.

*ING5* mRNA level was lower in ovarian cancer than normal ovary, and borderline than benign tumors (*p* < 0.05), and inversely linked to the differentiation of ovarian cancer (Figure 7A, *p* < 0.05). According to TCGA dataset, it was negatively associated with vascular invasion, lymphatic invasion, and FIGO staging of ovarian cancer (Figure 7B, *p* < 0.05). According to Kaplan–Meier plotter, there was a negative correlation between *ING5* mRNA expression and the overall or progression-free survival time of the patients with Grade 2, Grade 3, and Stage I cancer (Figure 7B, *p* < 0.05). ING5 protein was less expressed in primary cancer than normal ovary by Western blot (Figure 7C, *p* < 0.05).

Immunostaining revealed that ING5 expression was strong in ovarian adenoma, borderline tumor, adenocarcinoma and endometriod carcinoma, but not or very weak in normal fiber (Figure 7D). As shown in Table 1, cytoplasmic ING5 expression was stronger in primary cancer than that in normal tissue (*p* < 0.05), but weaker than the metastatic cancer (Table 1, *p* < 0.05). Nuclear ING5 expression was higher than benign and borderline tumors than in the normal ovary and primary cancer (Table 1, *p* < 0.05). Regardless of cytoplasmic or nuclear distribution, ING5 was less expressed in serous and mucinous adenocarcinoma than miscellaneous subtypes, and positively correlated with dedifferentiation and ki-67 expression of ovarian cancers (Table 2, *p* < 0.05).

**Table 1:** ING5 expression in ovarian epithelial carcinogenesis

Groups		Cytoplasmic ING5 expression					Nuclear ING5 expression				
	*n*	−	+	++	+++	%	−	+	++	+++	%
Normal ovary	73	48	8	13	4	32.9	48	9	13	3	32.9^**^
Ovarian benign tumor	118	76	32	6	4	35.6^#^	71	32	9	6	39.8^**^
Ovarian borderline tumor	39	21	6	7	5	46.2	20	9	5	5	48.7
Ovarian cancer	264	95	74	62	33	64.0^*#^	132	33	52	47	50.0
Metastatic cancer in omentum	36	12	13	11	0	66.7^#^	24	2	7	3	33.3

Abbreviations: PR, positive rate

^*^, compared with normal ovary, *p* < 0.05; ^#^, compared with ovarian benign tumor; ^**^, compared with benign tumor, *p* < 0.01.

**Table 2:** Relationship between ING5 expression and clinicopathological features of ovarian cancer

Clinicopathological features		Cytoplasmic ING5 expression						Nuclear ING5 expression					
	*n*	-	+	++	+++	%	*p* value	-	+	++	+++	%	*p* value
Age (years)							0.029						0.334
< 56	139	45	33	41	20	67.6		65	19	29	26	53.2	
≥ 56	125	50	41	21	13	60.0		67	14	23	21	46.4	
Pathological classification							0.001						0.012
Serous adenocarcinoma	158	67	43	34	14	57.6		91	13	27	27	42.4	
Mucinous adenocarcinoma	20	10	6	4	0	50.0		12	1	6	1	40.0	
Miscellaneous subtypes	83	18	24	23	18	78.3		28	19	18	18	66.3	
FIGO staging							0.516						0.358
I–II	65	33	18	12	2	49.2		41	10	7	7	36.9	
III–IV	108	57	36	13	2	47.2		77	9	11	11	28.7	
Differentiation							< 0.001						0.001
Well-differentiated	31	18	8	3	2	41.9		18	8	4	1	41.9	
Moderately-differentiated	94	44	28	14	8	53.2		58	14	11	11	38.3	
Poorly-differentiated	109	32	26	33	18	70.6		44	18	25	27	59.6	
Ki-67 expression							0.100						0.001
−	50	27	14	9	0	46.0		42	3	4	1	16.0	
+	35	19	13	2	1	45.7		24	4	4	3	31.2	
++	33	18	9	5	1	45.5		22	6	1	4	33.3	
+++	35	12	14	8	1	65.7		18	4	7	6	48.6	

## DISCUSSION

ING5 suppressed growth and metastasis of lung cancer cells [11], promoted cell apoptosis and restricted proliferation of hepatocellular carcinoma cells [12, 16], and inhibited cell migration, invasion, and EMT of breast cancer cells [17]. Reportedly, ING5 suppressed bladder cancer chemoresistance and DNA damage response pathway [18]. In line with other reports [12, 13] , we found that ING5 overexpression suppressed cell proliferation, tumor growth, glucose and lipid metabolism, and induced cell cycle arrest, apoptosis, senescence, and autophagy of ovarian cancer cells, even highly-invasive or paclitaxel-resistant ones. Taken together, these studies indicated that ING5 might reverse the aggressive phenotypes of various cancer cells and be employed as a potential target of gene therapy.

Previous data showed that ING5 decreased the capability of invasion and migration in lung [11], breast [16] and colorectal [10] cancers. Here, ING5 overexpression resulted in less spindle-like fibroblastic structures and smaller nucleus with N-cadherin hypoexpression and E-cadherin overexpression, indicating that ING5 inhibited cell migration and invasion by reversing EMT process. Chen al. [19] reported that activated Akt caused loss of cell-cell adhesion, induction of cell motility, and changes in the expression or the distribution of various epithelial or mesenchymal markers. Zhao et al. [17] found that ING5 significantly inhibited the phosphorylation of PI3K and Akt in breast cancer cells, leading to MET. In addition, EMT is positively associated with aberrant activation of Wnt or the PI3K/Akt pathway, which activates GSK-3β and stabilizes β-catenin [20, 21]. We found that PI3K, Akt, and β-catenin were decreased in ING5 transfectants of ovarian cancer cells. It was suggested that ING5 might weaken PI3K/Akt signal pathway and then down-regulate β-catenin, finally to suppress EMT. Furthermore, ING5-mediated down-regulation of MMP-2, MMP-9 and VEGF accounted for anti-invasion and anti-metastasis effects of ING5 because they promoted the degradation of extracellular matrix and angiongenesis [22].

As report goes, BCRP, LRP1 and MRP1 proteins act as pump transporter in multiple drug resistance, whereas GST-π can degrade the drugs by reducing reaction [23]. FBXW7 silencing mediated the chemoresistance of cancer cells [24]. Activation of NF-κB signaling up-regulates transcription of Bcl-xL, and subsequently mediates chemoresistance [25]. CD147 can assembly or stabilize signaling and transporter complexes within specialized lipid raft, containing EGFR, ABC-family drug transporters, which are responsible for anti-apoptosis and chemoresistance [26]. Our results hinted that the ING5-mediated drug resistance might be due to the hyperexpression of MRP, GST-π, MDR1, BCRP, CD147, NF-кB and Bcl-xL, and the hypoexpression of FBXW7.

Reportedly, Bcl-2 can interact with Bax on the mitochondrial membrane to suppress the apoptosis, which is prevented by the complex formation of phosphorylated BAD and 14-3-3 [27, 28]. Nuclear p53 can promote the transcription of Bax [29]. After phosphorylation by Akt, BAD is demonstrated to be released from Bcl-2 and loses its pro-apoptotic effect [30]. Here, lower expression of Bcl-2 and p-Akt, and higher expression of p53, Bax and 14-3-3 accounted for the apoptotic induction of ING5 via mitochondrial pathway. ATG13-ULK1-RB1CC1 complex, LC-3B and Beclin 1-UVRAG-VPS34-Ambra 1-ATG14 complex were involved in autophagy formation [31]. Our findings demonstrated that ING5 up-regulated LC-3B, Beclin 1 and ATG13 expression with autophagy strengthened in ovarian cancer cells, indicating that ING5 induced autophagy in Beclin 1-dependent manner.

Hexokinase I (HXKI) converses glucose to glucose-6-phosphate [32] and phospho- fructokinases 1(PFK-1) converts fructose 6- phosphate into fructose 1, 6-bisphosphate [33] during glycolysis. Citrate synthase (CS) catalyzes acetate residue and oxaloacetate to form the citrate in tricarboxylic acid cycle [34]. The inhibitory effects of ING5 on glucose metabolism were positively linked to HXK1, PFK-1 and CS hypoexpression. Adipophilin (ADFP) is a ubiquitous component of lipid droplets and is a useful marker for lipid droplet accumulation [35]. Aberrant fat accumulation in ING5 transfectants might be attributable to the up-regulatory effect of ING5 on ADFP expression in ovarian cancer cells.

Previously, nuclear ING5 shift to the cytoplasm was observed in the tumorigenesis of gastric [9], colorectal [10], breast [36], head and neck squamous carcinoma [8] respectively. Our results showed that ING5 level was lower in ovarian cancer than normal ovary at both mRNA and protein, while cytoplasmic and nuclear ING5 expression was immunohistochemically increased from normal ovary, ovarian benign and borderline tumors to cancer. The paradoxical phenomenon could also be explained by the presence of ING5 expression in stromal cells and the different karyoplasmic ratio. ING5 expression was lower in serous and mucinous adenocarcinoma than endometrioid and clear cell carcinoma, indicating that its close link with the histogenesis of the latter two subtypes. It was positively associated with dedifferentiation of ovarian cancer, opposite to the *in vivo* and vitro data of gastric cancer [9].

Previously, nuclear ING5 expression was positively correlated with the favorable prognosis of the patients with gastric cancer [9] and lung cancer [11]. In breast cancer, *ING5* mRNA expression was positively with relapse- and distant metastasis-free survival rates [36]. In the present study, a negative correlation was demonstrated between *ING5* mRNA expression and the overall or progression-free survival time of the patients with Grade 2, Grade 3, and stage I cancer, but there was a negative association of ING5 mRNA expression with local invasion and clinical staging. The discrepancy might be due to the findings of different datasets. These findings indicated that *ING5* mRNA might be used to evaluate the aggressiveness and prognosis of the ovarian cancer patients.

In short, ING5 overexpression suppressed the proliferation, energy metabolism, migration, invasion and tumor growth, but induced apoptosis, autophagy, senescence, and drug resistance of ovarian cancer cells. Our study hinted that altered ING5 expression might impact on the malignant transformation of ovarian cancer cells and should be identified with a good biomarker for ovarian carcinogenesis. Therefore, ING5 should be considered as a novel biomarker for ovarian carcinogenesis and a molecular target of gene therapy for ovarian cancer.

## MATERIALS AND METHODS

### Cell culture and treatment

Ovarian cancer cells were maintained in RPMI 1640 (ES-2, H08910, H08910-PM, OVCAR3, SKOV3/DDP, A2780 and A2780/T), DMED(CAOV3) and McCoy's 5A(SKOV3) medium supplemented with 10% fetal bovine serum, 100 units/mL penicillin, and 100 μg/mL streptomycin in a humidified 5% CO_2_ at 37°C. Some cells were treated with cisplatin (Hansoh Pharm, DDP), paclitaxel (Harbin Pharmaceutical Group Co., Ltd), MG132 (Enzo, proteosome inhibitor) and SAHA (Cayman Chem Com, a HDAC inhibitor).

HO8910, HO8910-PM, A2780 and A2780/T cells were transfected with pEGFR-N1-ING5 or pEGFP-N1 vector after seeding on dishes, selected by G418 according to the manufacturer’s instructions (QIAGEN). The autophagosome formation was detected by the localization of exogenous LC3B fused to enhanced green fluorescent protein (EGFP-LC3B). Briefly, the transfection of EGFP-LC3B plasmid was carried out by Lipofectamine LTX and PLUS^™^ reagent (Invitrogen).

### Immunofluorescence

Cells were seeded on glass coverslips until adhesion, the sections were fixed with paraformaldehyde (4% in PBS 1X) and permeabilized with Triton X-100 solution (0.1% in PBS 1X) for 10 min. Then the sections was blocked by 10% bovine serum albumin and then incubated with anti-ING5 antibody and then with Alexa Fluor^®^ 488 IgG at 4ºC. Then nuclei were stained with 1 μg/mL DAPI at 37ºC. Finally, coverslips were mounted with SlowFade^®^ Gold antifade reagent (invitrogen) and observed using laser confocal scanning microscope.

### MTT assay

Cell Counting Kit-8 (CCK-8) was employed to determine cell viability as instructions.

### Cell cycle analysis

The cells were trypsinized, collected, washed by PBS twice and fixed in cold 10 mL ethanol. Then, the cells were washed by PBS twice and incubated with 1mL RNase (0.25 mg/mL) at 37°C. The cells were pelleted, resuspended in propidium iodide (PI) at a concentration of 50 µg/mL and incubated at 4°C in the dark. Finally, flow cytometry was employed to examine PI signal.

### Annexin-V-FITC labeling and fluorescence-activated cell sorting analysis

PI and FITC-labeled Annexin V (Keygen, China) was used to detect membranous phosphatidylserine externalization as an indicator of apoptosis. PI is employed to differentiate viable from nonviable cells.

### Transwell migration and invasion assay

The migration and invasion assays were performed using Transwell chamber (Corning, Michigan, USA). Cells with serum-free culture medium were seeded into each well of the upper transwell chamber without or with biocoat matrigel (BD Biosciences). In the lower chamber, RPMI1640 with 10% fetal bovine serum was added. After incubating for 48 h, the cells were stained with crystal violet.

### Wound healing assay

Cells were plated in 6-well plate and allowed to grow to confluence. Medium was removed and wounds were introduced by scraping the confluent cell cultures with a 200 μL pipette tip. Floating cells were carefully removed before complete medium was added. The cells were incubated at 37°C. The wound healing process was monitored under an inverted light microscope.

### β-galactosidase staining

β-galactosidase staining was performed with a senescence-associated β-Galactosidase Staining Kit (Beyotime, China) according the recommended protocol.

### Oil red O staining

Ovarian cancer cells were cultured in 6-wells chamber slides. Cells were washed three times with PBS, and stained with oil red O according to previously described methods [36].

### Measurement of extracellular acidification and oxygen consumption rates

Seahorse metabolic flux analyser was used to measure the metabolic parameters in wing discs of abx UbxFLPase as previously reported [13].

### Xenograft models

Locally bred female Balb/c nude mice were used for implantation at 3–4 weeks. They were maintained under specific pathogen-free conditions, and food and water were supplied ad libitum. Housing and all procedures were performed according to protocols approved by the Committee for Animal Experiments guidelines on animal welfare of China Medical University. Subcutaneous xenografts were established by injection of cancer cells per mouse to axilla (*n* = 10 mice /group). Until tumor diameter reached 6 mm, we began to intraperitoneally inject 20 mg/kg SAHA into mice from 1th, 3th, and 5th day. For each tumor, measurements were made using calipers, and tumor volumes were calculated as follows: length × width ×depth× 0.52. After anesthetization and ultrasonic examination, the mice were photographed and sacrificed. The tumors were subjected to volume measurement and weighted. The part of tumors were subsequently fixed in 4% paraformaldehyde for 24 h, and then embedded in paraffin for following experiments.

### Subjects

Ovarian normal tissue, benign and borderline tumors, primary and metastatic cancers in omentum were collected from surgical resection at Shengjing Hospital of China Medical University between January 2005 and December 2011. The average age at surgery was 51.2 years (20–81 years). The parts of ovarian tissues were subjected to the routine preparation of pathological block. Some samples were frozen immediately in liquid nitrogen and stored at −80°C until use. None of the patients underwent chemotherapy, radiotherapy or adjuvant treatment before surgery. Informed consent was obtained from all subjects and the study protocol was approved by the Ethics Committee of our hospital.

### Quantitative RT-PCR

Total RNA was isolated using QIAGEN RNeasy mini kit. The first strand cDNA was synthesized using AMV reverse transcriptase and random primer (Takara). Oligonucleotide primers for PCR were shown in Supplementary Table 1. Real-time PCR was performed using SYBR Premix Ex Taq^®^ II kit (Takara). The expression level was expressed as 2^-∆Ct^, where ∆Ct = Ct (gene) – Ct (GAPDH). Additionally, the expression level of the control cells was considered as “1” in cell experiment.

### Western blot

Protein was extracted in RIPA lysis buffer by sonication, denatured, separated on SDS-polyacrylamide gel, transferred to Hybond membrane, and then blocked overnight in 5% skim milk in TBST. For immunoblotting, membrane was incubated with the primary antibodies Supplementary Table 2. Then, it was rinsed by TBST and incubated with the secondary antibodies for 2 h at room temperature. Bands were visualized with Fuji LAS4010 by ECL-Plus detection reagents (Santa Cruz). GADPH or β-actin was considered as internal control because they are house-keeping proteins. Densitometry quantification was performed with an internal control using Scion Image software. Additionally, the expression level of the control cells was considered as “1”.

### Immunohistochemistry (IHC)

IHC and its evaluation were carried out as previously reported [13]. The rabbit anti-ING5, anti-ki-67 and anti-LC-3B antibodies were purchased from Proteintech, DAKO and Santa Cruz respectively.

### TUNEL

Terminal deoxynucleotide transferase (TdT) mediated dUTP nick labeling (TUNEL) was performed using Apoptosis Detection Kit (Millipore, USA) according to the manufacturer’s instructions.

### Bioinformatics analysis

The expression data (RNA-seqV2) and clinicopathological data of 304 ovarian cancer patients were downloaded from the Cancer Genome Atlas (TCGA) database by TCGA-assembler in R software. We integrated the raw data, analyzed ING5 expression in ovarian cancer, and compared it with clinicopathological and prognostic data of the patients with ovarian cancer. The prognostic significance of *ING5* mRNA was analyzed using Kaplan–Meier plotter (http://kmplot.com).

### Statistical analysis

Results are representative of 3 different experiments, and data are expressed as mean ± standard deviation. *Spearman’s* correlation test was performed to analyze the rank data, and Mann-Whitney U to differentiate the means of different groups. SPSS 10.0 was applied to analyze all data and *p* < 0.05 was considered statistically significant.

## SUPPLEMENTARY MATERIALS TABLES


