# Supplementary material for: The roles of ING5 expression in ovarian carcinogenesis and subsequent progression: a target of gene therapy
Source: Oncotarget. 2017 Oct 19;8(61):103449–64. doi: 10.18632/oncotarget.21968 (PMC5732741; doi:10.18632/oncotarget.21968)
Supplement: Supplementary file 1 [file oncotarget-08-103449-s001.pdf]

The roles of ING5 expression in ovarian carcinogenesis and subsequent progression: a target of gene therapy

SUPPLEMENTARY MATERIALS

Supplementary Table 1: Primers employed in the present study

Names	Primer‘ s sequence	Distribution	AT (°C)	Product size (bp)	Extension time (s)
E-cadherin	F:5′-CCGCCATCGCTTACA-3′<br>R:5′-GGCACCTGACCCTTGTA-3′	NM-057374.2<br>1017-1278	60	262	34
N-cadherin	F:5′-GAAAGACCCATCCACG- 3′<br>R: 5′-CCTGCTCACCACCACTA- 3′	NM-031333.1<br>2365-2581	60	217	34
MMP2	F:5′- TGATCTTGACCAGAATACCATCGA- 3′<br>R: 5′- GGCTTGCGAGGGAAGAAGTT - 3′	XM_004057658.2<br>429-518	60	94	34
MMP9	F:5′- TGTACCGCTATGGTTACACT - 3′<br>R: 5′- CCTCAAAGGTTTGGAAT - 3′	NM_004994.2<br>169-353	60	189	34
MRP1	F:5′- TTTCAGAACACGGTCCTCG - 3′<br>R: 5′-TGGGCTGACCAGAAACACT - 3′	XM_017023243.1 154-387	60	234	34
TOPI	F:5′- AAAGATCGAGAACACCGG- 3′<br>R: 5′- TGTTTGGTCTTCTCCTTCT- 3′	XM_004062154.1<br>335-456	60	122	34
TOPII	F:5′-AAAATGAAGATGCTAAGAAAAGACT-3′<br>R:5′-GTACAAACCAGGAACAAAAGTGACT-3′	XM_004041770.2<br>218-405	60	188	34
FBXW7	F:5′-AGATGGACCAGGAGAGTG-3′<br>R: 5′-CTTGCATGGTTTCTTTCC-3′	NM_001349798.1 1531-1748	60	218	34
VEGF	F:5′- GCGCTCGGTGCTGGAATTTG -3′<br>R: 5′- TAGAGCAATCTCCCCAAGCCG -3′	XM_016955597.1<br>138-298	60	161	34
MDR1	F:5′- ACACCTGGGCATCG -3′<br>R: 5′- TATTAGGCAGTGACTCGA -3′	NM_001348946.1 3878-4035	60	158	34
BCRP	F:5′- GACAGCTTCCAATGACCTGAA -3′<br>R: 5′-CAGGATGGCGTTGAGACC -3′	NM_001348989.1 204-375	60	172	34
GST-π	F: 5′- CGGGCAAGGATGACTATGTGA -3′<br>R: 5′- GGGCTAGGACCTCATGGATCA -3′	BC044846.1<br>1137-1475	60	339	34
GAPDH	F: 5′-CAATGACCCCTTCATTGACC-3′<br>R: 5′- TGGAAGATGGTGATGGGATT-3′	NM_ 002046.3<br>201-335	60	135	34

AT = annealing temperature.

Supplementary Table 2: The antibodies used for western blot and immunohistochemistry

Num	Antibody	Species	Dilution	Company	Code number
1	Bcl-2 (C-21)	rabbit	1:700	santa cruz	sc-783
2	β-catenin(E-5)	mouse	1:1000	santa cruz	sc-7963
3	LC-3B	rabbit	1:1000	wanleibio	wl01506
4	ADFP	rabbit	1:2000	abcam	ab51031
5	ING5	goat	1:1000	abcam	ab3716
6	LaminB(C-20)	goat	1:500	santa cruz	sc-624
7	Bax(B-9)	mouse	1:500	santa cruz	sc-7480
8	P53	rabbit	1:1000	wanleibio	WL02384
9	Cdc25b(C-20)	rabbit	1:500	santa cruz	sc-326
10	Cdk4 (C-22)	rabbit	1:500	santa cruz	sc-260
11	PI3 Kinase	rabbit	1:1000	abcam	ab151549
12	14-3-3(H-8)	mouse	1:500	santa cruz	sc-1657
13	Akt	rabbit	1:500	santa cruz	sc-8312
14	p-Akt	rabbit	1:500	santa cruz	sc-16646
15	Bcl-xs/l(S-18)	rabbit	1:500	santa cruz	sc-634
16	LRP	rabbit	1:1000	abcam	ab28320
17	BCRP	rabbit	1:1000	abcam	ab108312
18	CD147	rabbit	1:1000	abcam	ab108317
19	GSTπ	rabbit	1:1000	abcam	ab34934
20	NFKB	rabbit	1:1000	CST	8242s
21	MMP-9(2C3)	mouse	1:500	santa cruz	sc-21733
22	MMP-2	mouse	1:500	santa cruz	sc-13594
23	N-cadherin	mouse	1:1000	abcam	ab98952
24	ATG13	rabbit	1:1000	abcam	ab155826
25	HXK1(N-19)	goat	1:500	santa cruz	sc-6517
26	CS(P-20)	goat	1:500	santa cruz	sc-242444
27	E-cadherin	mouse	1:1000	abcam	ab76055
28	VEGF(A-20)	rabbit	1:500	santa cruz	sc-152
29	Beclin 1	rabbit	1:500	santa cruz	sc-11427
30	Ki-67	rabbit	1:300	abcam	Ab15580
31	GAPDH(6C5)	mouse	1:2000	santa cruz	sc-32233
32	β-actin(C4)	mouse	1:2000	santa cruz	sc-47778
